# Effects of spirulina powder on the physicochemical and volatile properties of rice bahlls

**DOI:** 10.1016/j.fochx.2026.104141

**Published:** 2026-06-25

**Authors:** Xiwu Jia, Yujun Shi, Zhili Ji, Wangyang Shen, Xin Liu, Bo Yu, Yolani Syaputri, Hongjian Zhang

**Affiliations:** a Department of Food Science and Engineering, Wuhan Polytechnic University, Wuhan 430023, China; b Key Laboratory for Deep Processing of Major Grain and Oil, Ministry of Education, Wuhan Polytechnic University, Wuhan 430023, China; c Hainan Institute of Grain and Oil Science, Qionghai 571400, China; d School of Food Science and Chemical Engineering, Hubei University of Arts and Science, Hubei, Xiangyang, 441053, China; e Biology Department, Universitas Padjadjaran, Bandung, Indonesia

**Keywords:** Spirulina powder, Rice ball, Physicochemical properties, Microstructure, Volatile flavor compounds

## Abstract

This study compared the quality of rice balls prepared with different concentrations of spirulina powder (0%, 1%, 3%, 5%, and 7%) and investigated their nutritional composition, color parameters, hardness, water distribution, and fourier transform infrared spectroscopy (FTIR), X-ray diffraction (XRD), scanning electron microscopy (SEM), and gas chromatography-ion mobility spectrometry (GC-IMS) findings. Spirulina significantly improved the nutritional value of rice balls, with the protein content increasing by 38.37%. With increasing spirulina concentrations, the L* and W values of rice balls decreased, causing a green appearance with progressively intensified color, whereas the hardness increased significantly. Low-field nuclear magnetic resonance (LF-NMR) findings revealed that low spirulina concentrations (1%–5%) increased the proportion of bound water and decreased that of free water, whereas at 7% concentration, T_22_ increased and the proportion of free water rebounded, indicating a reduced water-binding capacity of the system. The FTIR and XRD results indicated that spirulina altered the short-range molecular order and long-range crystalline structure of starch. Furthermore, R_1049/1022_ and the relative crystallinity increased by 17.53% and 18.5%, respectively, with increasing spirulina concentrations, indicating that spirulina accelerated short-term starch retrogradation. SEM observations further indicated that spirulina addition disrupted the continuous starch matrix, resulting in enlarged pores and an increased pore number. GC-IMS identified 52 volatile compounds, and spirulina significantly altered both the composition and relative abundance of aroma-active compounds. These data provide a theoretical basis for developing nutrient-enriched rice-based products.

## Highlights

• Spirulina increased the protein and mineral content of rice balls.
• Spirulina changed rice ball color, hardness, and water-binding properties.
• Spirulina enhanced starch ordering and accelerated retrogradation.
• Spirulina disrupted the starch matrix and modified microstructure
• Spirulina reshaped the volatile profile and relative abundance of rice balls.

## Graphical abstract

**Figure:**
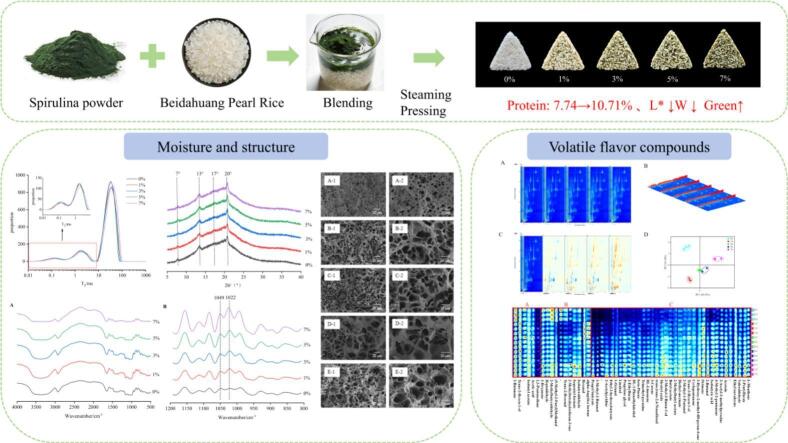

Permanent address: Hainan Institute of Grain and Oil Science, Qionghai 571,400, China.

## 1 Introduction

Rice balls are prepared by pressing cooked rice into a mold, and various ingredients can also be added to enrich their texture (Binti et al., 2024). Rice balls can be classified according to their shape into triangular, cylindrical, spherical, and other forms. With economic development and increasingly fast-paced lifestyles, rice balls have gained attention as a convenient rice-based food with promising market potential. However, conventional rice balls are generally prepared from polished rice and are therefore characterized by a starch-dominated nutritional profile. Although they provide a convenient source of dietary energy, their contents of protein, dietary fiber, minerals, and bioactive compounds are relatively limited. Previous studies have shown that rice polishing removes the bran and germ layers, resulting in substantial losses of dietary fiber, lipids, minerals, vitamins, and phytochemicals (Saleh et al., 2019; Shobana et al., 2011). In addition, lysine is the limiting amino acid in rice protein, which further restricts the nutritional balance of rice-based foods (Jayaprakash et al., 2022). Therefore, improving the nutrient density of rice balls through suitable food fortification is important for developing value-added rice-based staple foods. On the other hand, rice balls are primarily composed of starch, and their quality depends on starch gelatinization and retrogradation as well as water migration. Current research on inhibiting starch retrogradation primarily focuses on factors such as the intrinsic composition of raw materials, the surrounding environmental conditions, added exogenous ingredients, physical modification techniques, and processing parameters (Chang et al., 2021; Hirata et al., 2025; Lu et al., 2025). Among these approaches, the incorporation of exogenous additives has received considerable attention because it is simple to implement, highly adaptable, and amenable to industrial application, thus providing a feasible route for developing nutritionally fortified rice-based staple foods.

Spirulina is a filamentous, multicellular cyanobacterium characterized by rapid growth and the advantage of not competing with staple crops for arable land (Maleterova et al., 2015), which make it one of the most widely used and commercially mature microalgal resources. Due to its rich content of essential nutrients, including high-quality plant protein, vitamins, carotenoids, and various fatty acids, spirulina is considered a valuable dietary supplement. It is produced on a large scale worldwide and has been widely applied in animal feed, pharmaceuticals, and food products (Luo et al., 2024; Withana et al., 2025). It provides multiple health benefits, including anti-inflammatory, antioxidant, anticancer, hypolipidemic, hypoglycemic, and immunomodulatory activities, and is generally considered to exert no significant adverse effects (Hatami et al., 2021).

The incorporation of spirulina can improve the nutritional value of the product, improve its elasticity and overall mouthfeel, and impart a distinctive color and flavor (Luo et al., 2024). For instance, Veena et al. (2022) reported that the addition of spirulina promoted the formation of a protein–starch network, enabling better dispersion of starch granules, improving the water absorption and swelling of pasta, and prolonging the optimal cooking time. Çakır et al. (2024) also reported that incorporating spirulina powder (0.5%–5%) into rice flour biscuits significantly improved their nutritional value; however, increasing the addition level darkened the product color and increased the hardness and brittleness, causing a decline in sensory acceptability, with the 0.5% formulation receiving the highest preference score. With the increasing emphasis on improving the nutritional quality of staple foods, spirulina powder has been extensively investigated in wheat-based products, whereas studies on its application in rice-based foods remain relatively limited. Therefore, based on previous comparative studies on the effects of different addition levels of chlorella and spirulina on the physicochemical properties, structure, and rheological performance of rice starch (Jia et al., 2025), this study was conducted to further clarify the effects of spirulina powder addition on the nutritional value, physicochemical properties, microstructure, and volatile profile of rice balls, thereby providing a theoretical basis for the utilization of spirulina powder in rice-based products.

## 2 Materials and methods

### 2.1 Materials and chemicals

Rice was purchased from Beidahuang Food Group Co., Ltd. (Harbin, China). Food-grade spirulina powder was obtained from Fuqing King Dnarmsa Spirulina Co., Ltd. (Fuzhou, China). The basic compositions of rice and spirulina powder are presented in Table 1. All other chemicals used in this study were of analytical grade.

**Table 1:** Basic components of rice and spirulina powder.

Sample	Protein (%)	Fat (%)	Total starch (%)	Amylose (%)	Dietary fiber (%)	Ash content (%)
Rice	7.94 ± 0.05	0.75 ± 0.05	77.80 ± 0.99	17.23 ± 0.03	1.63 ± 0.07	0.39 ± 0.03
Spirulina powder	63.44 ± 0.09	5.70 ± 0.70	–	–	5.55 ± 0.52	5.81 ± 0.09

### 2.2 Sample preparation

Rice samples of 150 g were rinsed three times with ultrapure water within 3 min. Subsequently, spirulina powder was incorporated into the pre-rinsed rice samples in dry state at mass ratios of 1%, 3%, 5%, and 7% (*w*/w), and the mixtures were fully blended to achieve uniform distribution. Afterwards, ultrapure water was supplemented at a rice-to-water ratio of 1:1.3 (*v*/v), and the resultant mixtures were steeped at 20 °C for 30 min. The rice was then cooked in a rice cooker (SR-DB071-W, Panasonic Kitchen Appliances Co., Ltd., Hangzhou, China) using the steaming mode (400 W) for 40 min, after which it was stirred twice in both horizontal and vertical directions using chopsticks, covered, and maintained warm for 15 min. Finally, 90 g of the cooked rice was molded to form rice balls and then wrapped in a plastic wrap.

### 2.3 Determination of the basic composition of raw materials

The moisture, total ash, fiber, and fat contents of rice and spirulina powder were determined using standard AOAC methods (Aoac, 2005). The crude protein content was determined using a Vario EL Cube elemental analyzer (Elementar, Langenselbold, Germany) according to a modified method of Krotz et al. (2016). Briefly, 5 mg of each sample was accurately weighed, wrapped in tin foil, compacted, and placed into the sample tray. Elemental analysis was performed using sulfadiazine as the calibration standard according to the instrument operating program. The carbohydrate content was estimated using the difference (100 − ∑ other components). The total starch content was determined using a Total Starch Assay Kit (K-TSTA-100 A, Megazyme, Bray, Ireland) (Murai & Annor, 2025). The amylose content was determined using an assay kit (BC4265, Solarbio, Beijing, China) based on the iodine colorimetric method (Xia et al., 2024).

### 2.4 Color measurement

The color of the rice ball was evaluated using a colorimeter (CS-10, Caipu Technology Co., Ltd., Zhejiang), and the L*, a*, and b* values were recorded. Next, the whiteness value (W) and the color difference value (∆E) were calculated according to the following equation:

$$W=100-\sqrt{{\left(100-L^{*}\right)}^{2}+{a^{*}}^{2}+{b^{*}}^{2}}$$

$$\mathrm{\Delta E}=\sqrt{{\left(\Delta L^{*}\right)}^{2}+{\left(\Delta a^{*}\right)}^{2}+{\left(\Delta b^{*}\right)}^{2}}$$

where ∆L*, ∆a*, and ∆b* represent the differences between the corresponding color parameters of the sample and control (without spirulina powder addition).

### 2.5 Texture measurement

Texture profile analysis (TPA) of prepared samples was conducted following the protocol established by Tao et al. (2020). Briefly, an 8 g rice ball sample was transferred into a stainless-steel cylindrical mold with an inner diameter of 30 mm and a height of 9 mm. Subsequently, the assembled sample was positioned on the testing platform of a texture analyzer equipped with a P/36R cylindrical probe. The instrumental parameters were set as follows: pre-test speed, 1.00 mm/s; test speed, 1.00 mm/s; post-test speed, 2.00 mm/s; trigger force, 1.5 N; compression deformation rate, 50.0% of the initial sample height; and interval time between two compression cycles, 5.00 s. Texture indices including hardness, cohesiveness and springiness were automatically acquired during testing. All measurements were performed in five independent replicates.

### 2.6 LF-NMR determination

Moisture distribution within rice ball samples was characterized via LF-NMR according to the method of Horigane et al. (2013) with minor modifications. Transverse relaxation time (*T*_2_) was determined using an LF-NMR spectrometer (MicroMR-CL-I, Suzhou Niumag Analytical Instrument Corporation, Suzhou, China). Briefly, approximately 5 g of rice ball sample was sealed with plastic film and placed into a 40 mm diameter NMR tube prior to measurement. The experimental parameters were as follows: spectral frequency (SF), 20 MHz; offset frequency (O_1_), 679381.68 Hz; sampling frequency (SW), 200 kHz; number of sampling points (TD), 79996; sampling interval time (TW), 2000 ms; number of echoes (NECH), 4000; echo time (TE), 0.100 ms; and number of scans (NS), 8.

### 2.7 FTIR analysis

The short-range ordered structure was measured as described by Tu et al. (2021), with slight modifications. Briefly, vacuum freeze-dried samples were ground into rice flour and mixed with potassium bromide at a 1:100 (*w*/w) ratio and ground into a fine powder using an agate mortar and pestle. After uniform grinding, the mixture was pressed into pellets using a tablet press. The prepared pellets were analyzed using an FTIR spectrometer (Frontier, PerkinElmer, USA) over the range of 400–4000 cm^−1^, with a total of 32 scans. Before sample analysis, an air background scan was performed to eliminate interference. Fourier self-deconvolution was performed using the OMNIC software, and the absorbance ratio at 1049/1022 cm^−1^ was used to determine the short-range order of starch in the rice balls.

### 2.8 XRD analysis

The crystalline properties of rice balls were evaluated as described by Zhang et al. (2022), with slight modifications. After passing the samples described in Section 2.7 through a 200-mesh sieve, they were scanned using an X-ray diffractometer (D8 Advance, Bruker, Germany). The diffraction angle range was 5°–40°, with a scanning rate of 5° min^−1^. The X-ray source used Cu Kα radiation (λ = 0.154056 nm). The areas of the amorphous region (A_a_) and crystalline region (A_c_) were calculated using the Origin 9.0 software. The relative crystallinity (RC) was calculated using the following equation:

$$\mathrm{RC}=\frac{A_{c}}{A_{c}+A_{a}}\times 100\%$$

### 2.9 SEM analysis

The microstructure of rice balls was evaluated according to Bui et al. (2018), with slight modifications. After vacuum freeze-drying the samples, intact rice kernels were gently fractured at the middle perpendicular to the long axis using a blade, and the ends were removed to obtain cross-sections approximately 3 mm thickness as scanning samples. The samples were then observed using the Gemini SEM 300 instrument (Carl Zeiss Ltd., Jena, Germany). The samples were mounted on a conductive tape, gold-coated, and observed under a scanning electron microscope at an accelerating voltage of 3 kV.

### 2.10 Volatile properties

The volatile components in rice balls were identified using a gas-phase ion migration spectrometer (1H1–00053, G.A.S., Germany). Rice ball samples (2 g) were placed in a 20 mL headspace vial and incubated at 80 °C with agitation at 500 rpm for 20 min. The headspace syringe temperature was set at 85 °C, and the injection volume was 500 μL. Nitrogen was used as both carrier and drift gas. The carrier gas flow rate was programmed as follows: 0–2 min, 2 mL/min; 2–10 min, 2–10 mL/min; 10–20 min, 10–100 mL/min; and 20–30 min, 100–150 mL/min. The IMS detector temperature was set at 45 °C, and the total analysis time was 30 min. Qualitative analysis of volatile compounds was performed using GC × IMS Library Search, which incorporates the built-in NIST 2014 and IMS databases.

### 2.11 Statistical analysis

All data are expressed as the mean ± standard deviation of triplicate samples (unless otherwise specified). Data were organized and calculated using the Excel 2013 software, plotted using the Origin 9.0 software, and analyzed for significant differences using Duncan's multiple range test in the SPSS 26.0 software. *P* < 0.05 was considered statistically significant.

## 3 Results and discussion

### 3.1 Nutritional composition

The effects of adding different concentrations of spirulina powder on the nutritional composition of rice balls are presented in Table 2. The addition of spirulina powder at various concentrations (0%, 1%, 3%, 5%, and 7%) to the rice balls significantly increased the protein and ash contents (*P* < 0.05) in a concentration-dependent manner. The fat and dietary fiber contents also demonstrated an increasing trend, whereas the carbohydrate content decreased significantly (P < 0.05). Similar results have been reported in previous studies (Hussein et al., 2023; Koli et al. 2022). These findings were attributed to the relatively higher contents of protein, ash, fat, and dietary fiber in spirulina powder than in rice. Hence, the prepared rice balls may serve as functional foods rich in protein and minerals.

**Table 2:** Effects of adding different concentrations of spirulina powder on the nutritional composition of rice balls (dry basis).

Addition (%)	Protein (%)	Fat (%)	Carbohydrate (%)	Dietary fiber (%)	Ash (%)
0	7.74 ± 0.14^a^	0.68 ± 0.11^a^	89.76 ± 0.04^e^	1.54 ± 0.17^a^	0.33 ± 0.00^a^
1	8.23 ± 0.00^b^	0.79 ± 0.15^a^	89.03 ± 0.27^d^	1.56 ± 0.13^a^	0.38 ± 0.01^b^
3	8.82 ± 0.18^c^	0.85 ± 0.02^a^	88.14 ± 0.28^c^	1.74 ± 0.07^ab^	0.45 ± 0.01^c^
5	9.27 ± 0.18^d^	0.89 ± 0.09^a^	87.49 ± 0.19^b^	1.82 ± 0.09^ab^	0.52 ± 0.01^d^
7	10.71 ± 0.18^e^	0.92 ± 0.03^a^	85.78 ± 0.07^a^	1.94 ± 0.08^b^	0.66 ± 0.02^e^

Mean ± SD (*n* = 3); values in the same column with different letters are significantly different (*P* < 0.05) according to Duncan's test.

### 3.2 Color properties of rice balls

**Fig. 1:**
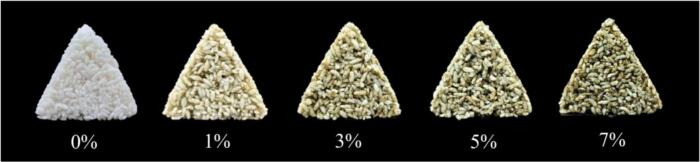
Appearance of rice balls prepared by adding different concentrations of spirulina powder (0%, 1%, 3%, 5%, and 7%).

The color properties of rice balls with the addition of different concentrations of spirulina powder (0%, 1%, 3%, 5%, and 7%) are depicted in Fig. 1 Appearance of rice balls prepared by adding different concentrations of spirulina powder (0%, 1%, 3%, 5%, and 7%)Adding spirulina powder to the rice balls resulted in a green appearance, with the color deepening gradually as the concentration increased. The color of the rice balls with the addition of different concentrations of spirulina powder are reported in terms of lightness (L*), redness (a*), yellowness (b*), whiteness (W), and total color difference (∆E) (Table 3 Effect of spirulina powder on the color of rice balls). Based on the magnitude of the color difference value ∆E, it is evident that the color difference between rice balls containing spirulina powder and blank rice balls was highly significant (*P* < 0.05). With increases in the concentration of spirulina powder, the brightness (L*) and whiteness (W) of the rice balls decreased significantly (P < 0.05), indicating a clear darkening trend.

**Table 3:** Effect of spirulina powder on the color of rice balls.

Addition (%)	L*	a*	b*	W	ΔE
0	70.46 ± 0.75^e^	−5.81 ± 0.14^e^	2.04 ± 0.04^a^	69.83 ± 0.75^e^	0
1	58.20 ± 1.23^d^	−7.84 ± 0.21^b^	6.24 ± 0.03^c^	57.01 ± 1.16^d^	13.13 ± 1.11^a^
3	50.73 ± 1.63^c^	−8.31 ± 0.27^a^	7.02 ± 0.05^d^	49.54 ± 1.64^c^	20.51 ± 1.60^b^
5	46.88 ± 0.07^b^	−6.68 ± 0.17^c^	6.15 ± 0.20^c^	46.11 ± 0.09^b^	23.95 ± 0.07^c^
7	43.92 ± 0.89^a^	−6.32 ± 0.09^d^	5.54 ± 0.09^b^	43.29 ± 0.88^a^	26.78 ± 0.87^d^

Mean ± SD (*n* = 3); values in the same column with different letters are significantly different (*P* < 0.05) according to Duncan's test.

The a* value exhibited a decreasing trend followed by an increase, whereas the b* value demonstrated an increasing trend followed by a decrease, which is consistent with the findings reported by Özyurt et al. (2015). These data indicated that when the spirulina concentration was in the range of 0%–3%, the rice balls tended to be greener and more yellow, whereas at 5%–7% concentrations, the rice balls tended to be redder and bluer. Arslan et al. (2021) reported that the important pigments in spirulina included chlorophyll *a*, β-carotene, astaxanthin, lutein, phycocyanin, and phycoerythrin, and the changes in the a* and b* values of the rice balls were primarily attributed to these pigments.

### 3.3 Textural properties of rice balls

The hardness of rice balls significantly increased (P < 0.05) with the addition of higher concentrations of spirulina powder (Table 4), indicating that spirulina powder contributed to a firmer texture. This change may be associated with enhanced starch retrogradation and structural ordering. The FTIR and XRD results showed that spirulina powder addition promoted the formation of short-range ordered structures and long-range crystalline structures in starch. This finding is consistent with that reported by Jia et al. (2025), who found that spirulina powder affected the gelatinization and retrogradation behavior of rice starch and promoted the structural ordering of rice starch gels. These results suggest that spirulina powder may promote the rearrangement and reassociation of starch molecular chains after gelatinization, thereby enhancing the structural strength of the rice ball system and increasing hardness. This finding is consistent with that reported by Marcinkowska-Lesiak et al. (2018), who reported that the addition of spirulina powder significantly increased the hardness of shortbread biscuits.

**Table 4:** Effects of adding different concentrations of spirulina powder on the textural properties of rice balls.

Addition (%)	Hardness (N)	Cohesiveness (N)	Springiness (N)
0	1.11 ± 0.05^a^	0.08 ± 0.02^a^	0.81 ± 0.01^a^
1	1.31 ± 0.05^b^	0.14 ± 0.01^c^	0.79 ± 0.02^a^
3	1.45 ± 0.10^c^	0.09 ± 0.01^a^	0.81 ± 0.02^a^
5	1.55 ± 0.05^c^	0.10 ± 0.02^ab^	0.80 ± 0.04^a^
7	1.73 ± 0.04^d^	0.12 ± 0.02^bc^	0.82 ± 0.02^a^

Mean ± SD (*n* = 5); values in the same column with different letters are significantly different (*P* < 0.05) according to Duncan's test.

The cohesiveness of rice balls demonstrated an inconsistent trend with the addition of different concentrations of spirulina powder. These findings revealed a higher cohesiveness of 1% Spirulina-fortified samples than that of the control sample, which could be attributed to the interaction between proteins and starch molecules that improved starch hydration. Meanwhile, the LF-NMR results showed that A_22_ increased while A_23_ decreased in the 1% group, indicating that part of the free water shifted toward immobilized water and that the water-fixing capacity of the system was enhanced, thereby contributing to the significant increase in the cohesiveness of rice balls. Furthermore, fibers interact with starch (Bai & Gilbert, 2022) and exhibit strong interactions with the protein–starch network (Nawaz et al., 2021), which can further improve the cohesiveness of the system. Nevertheless, with further increases in spirulina concentrations (3%–7%), the product cohesiveness exhibited a decreasing or fluctuating trend. This may be related to the increased introduction of non-starch components, such as proteins and dietary fibers, at higher spirulina powder levels. These components may compete for water and interfere with the continuous connections among gelatinized starch molecules, thereby reducing the structural cohesiveness of the rice ball matrix. Sissons et al. (2005) indicated that an increase in protein content can reduce the stickiness of pasta. In the present study, no significant differences were found in the springiness of any samples (*P* > 0.05), indicating that the addition of spirulina exerted no effect on the springiness of the rice balls.

### 3.4 Water distribution in rice balls

**Fig. 2:**
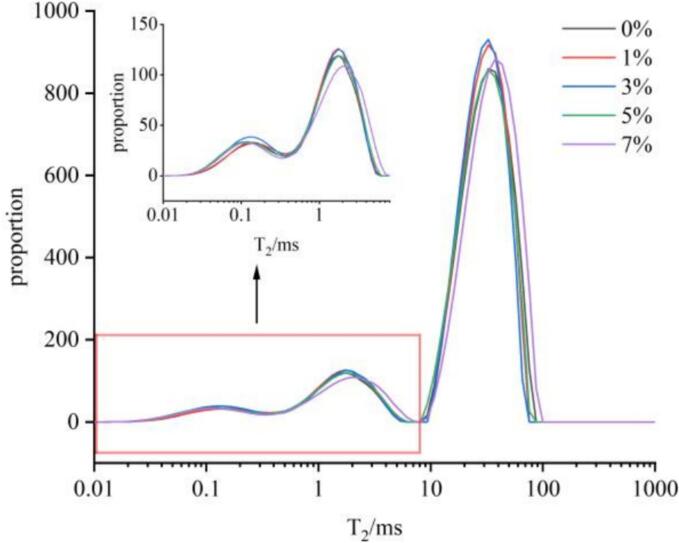
Transverse relaxation time (T_2_) distribution of rice balls.

The water content, distribution, mobility, and the manner by which water interacts with other components in starch-based foods exert a significant impact on their stability and functional properties (Chen et al., 2017; Jia et al., 2025; Luo et al., 2025). All the rice ball samples in the present study exhibited three characteristic T_2_ peaks in their relaxation spectra, corresponding to tightly bound water (T_21_), weakly bound water (T_22_), and free water (T_23_) (Fig. 2). With increasing concentrations of spirulina powder, no significant changes (*P* > 0.05) were detected in the T_21_ and T_23_ relaxation times of the rice balls. When the spirulina powder concentration reached 7%, the T_22_ relaxation time increased markedly (*P* < 0.05), indicating that the immobilized water in the rice balls became less restricted and more mobile. This suggests that a high level of spirulina powder may weaken the water-binding capacity of the system.

The values of A_21_, A_22_, and A_23_ represent the proportions of tightly bound water, weakly bound water, and free water, respectively (Table 5). With increases in the concentration of spirulina powder, the values of A_21_ and A_22_ first increased and then decreased, whereas the A_23_ value exhibited the opposite trend, decreasing initially and then increasing. When a small amount of spirulina powder is added, the proteins, polysaccharides, and dietary fibers bind part of the free water, which increases the levels of strongly and weakly bound water and, in turn, reduces the amount of free water in the system(Baranowska et al., 2008; Min et al., 2025; Xu et al., 2022; Yu et al., 2025). When the spirulina powder concentration reached 7%, the slight increase in A_23_ value, together with the marked increase in T_22_ value, indicated that the rice ball structure becomes looser at high spirulina concentrations. Consequently, the ability of the system to bind water decreases, resulting in a renewed increase in the proportion of free water.

**Table 5:** Effect of adding different concentrations of spirulina powder on water distribution in rice balls.

Addition (%)	T_21_/ms	T_22_/ms	T_23_/ms	A_21_/%	A_22_/%	A_23_/%
0	0.14 ± 0.02^a^	1.75 ± 0.00^a^	32.75 ± 0.00^a^	3.98 ± 0.69^a^	13.31 ± 0.51^ab^	82.70 ± 0.18^b^
1	0.16 ± 0.05^a^	1.75 ± 0.00^a^	32.75 ± 0.00^a^	4.61 ± 0.42^a^	13.69 ± 0.30^b^	81.71 ± 0.26^a^
3	0.12 ± 0.00^a^	1.75 ± 0.00^a^	32.75 ± 0.00^a^	4.68 ± 0.39^a^	13.89 ± 0.34^b^	81.43 ± 0.11^a^
5	0.13 ± 0.01^a^	1.75 ± 0.00^a^	32.75 ± 0.00^a^	4.45 ± 0.18^a^	13.69 ± 0.16^b^	81.86 ± 0.34^a^
7	0.14 ± 0.02^a^	2.21 ± 0.17^b^	37.65 ± 0.00^a^	4.43 ± 0.32^a^	12.73 ± 0.43^a^	82.84 ± 0.17^b^

Mean ± SD (*n* = 3); values in the same column with different letters are significantly different (*P* < 0.05). T_21_, T_22_, and T_23_ represent the relaxation times of strong bound water, weakly bound water, and free water, respectively. A_21_, A_22_, and A_23_ represent strong bound, bound, and free water contents, respectively.

### 3.5 Short-range order analysis

**Fig. 3:**
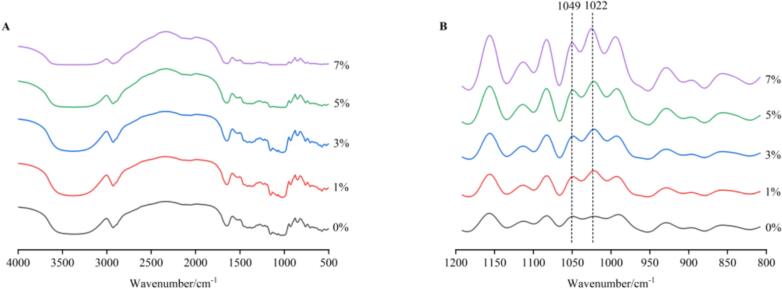
Fourier transform infrared (FTIR) spectroscopy (A) and deconvolution FTIR spectra (B) of the rice balls.

The short-range ordered structure of rice balls was investigated using an FTIR spectrometer, which revealed the double-helix structure of starch in short-range ordered reactions (Lu et al., 2021). The incorporation of spirulina powder did not generate any new characteristic absorption bands in the FTIR spectra (Fig. 3A), suggesting that no covalent bonding occurred between spirulina components and rice starch and that no new functional groups formed. Nevertheless, when the spirulina concentration was increased to 5%–7%, there was a marked change in the intensity of the broad band in the 3600–3000 cm^−1^ region, which was primarily attributed to intramolecular or intermolecular O—H stretching vibrations (Qin et al., 2020). This finding is primarily attributed to the high contents of proteins and polysaccharides in spirulina, which introduce abundant hydrophilic groups and improve the affinity for water, thus providing evidence for the results described in Section 3.4 regarding the increased mobility of bound and free water in rice balls.

The FTIR spectra were deconvoluted using the OMNIC software to obtain the deconvoluted spectra depicted in Fig. 3B. In the 1200–800 cm^−1^ region, the spectra exhibited changes associated with the short-range ordered structure of starch. Remarkably, the two dashed lines at 1049 and 1022 cm^−1^ correspond to the most critical characteristic bands for investigating the short-range order, where the band at 1022 cm^−1^ is related to the amorphous region of starch, and the band at 1049 cm^−1^ is associated with the short-range ordered structure of starch. Therefore, the ratio R_1049/1022_ is commonly used to characterize variations in the short-range order of starch (Zhang et al., 2023), with a higher R_1049/1022_ value indicating a greater degree of short-range molecular order and a higher extent of starch retrogradation (Zheng et al., 2024). In the present study, both the R_1049/1022_ value and the RC increased significantly with increasing spirulina powder concentrations (*P* < 0.05) (Table 6), indicating that incorporating spirulina powder improved the short-range order of starch structure in the rice ball system. The wet-heat treatment disrupts the starch structure; however, the addition of proteins not only improves the molecular order of starch but also strengthens the hydrogen bonding interactions between starch molecules (Wu et al., 2024). Shao et al. (2023) reported that an improvement in the short-range ordered structure of starch is associated with an increased protein content, and that proteins can, to some extent, promote the formation of short-range ordered structures in starch.

**Table 6:** Effects of adding different concentrations of spirulina powder on the short-range order and relative crystallinity of rice balls.

Addition (%)	R_1049/1022_	Relative crystallinity (%)
0	0.924 ± 0.010^a^	6.38 ± 0.19^a^
1	0.989 ± 0.011^ab^	6.92 ± 0.18^b^
3	1.017 ± 0.023^bc^	7.10 ± 0.09^bc^
5	1.062 ± 0.008^bc^	7.20 ± 0.07^c^
7	1.086 ± 0.133^c^	7.56 ± 0.11^d^

Mean ± SD (*n* = 3); values in the same column with different letters are significantly different (*P* < 0.05) according to Duncan's test.

### 3.6 Crystalline structure

**Fig. 4:**
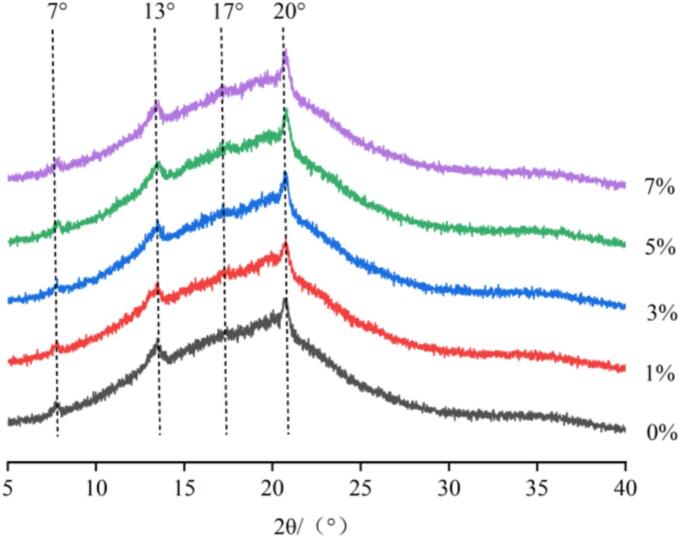
X-ray diffraction pattern of rice balls.

XRD is a typical method to determine the crystalline structure of starch-based materials. The XRD patterns of the rice balls with the addition of different concentrations of spirulina powder are depicted in Fig. 4. The sharp peaks represent crystalline regions, whereas the broad diffuse features correspond to amorphous regions. Diffraction peaks at 2θ ≈ 7°, 13°, and 20° were observed in all samples, which were primarily attributed to the formation of amylose–lipid complexes. In this complex, amylose binds with lipid molecules to form a single left-handed helical structure, known as the V-type crystal structure (Sun et al., 2020). In addition, a weak diffraction peak appeared at around 2θ ≈ 17°, the weak intensity of this diffraction peak may be due to the high moisture content of the rice balls, which restricted the rearrangement of starch chains and resulted in a relatively low degree of crystallinity (Liu et al., 2021). With increases in the concentration of spirulina powder, the V-type diffraction peaks at approximately 2θ ≈ 13° and 20° increased to some extent, indicating that higher spirulina powder concentrations could promote the formation of V-type crystallinity, which is consistent with the findings reported by Martínez-Sanz et al. (2018). The RC of the rice balls increased from 6.38% to 7.56% (Table 6), indicating an improvement in the long-range molecular order and a promotion of short-term retrogradation; macroscopically, this was manifested as a marked increase in the hardness of rice balls.

### 3.7 Microscopic structure analysis

**Fig. 5:**
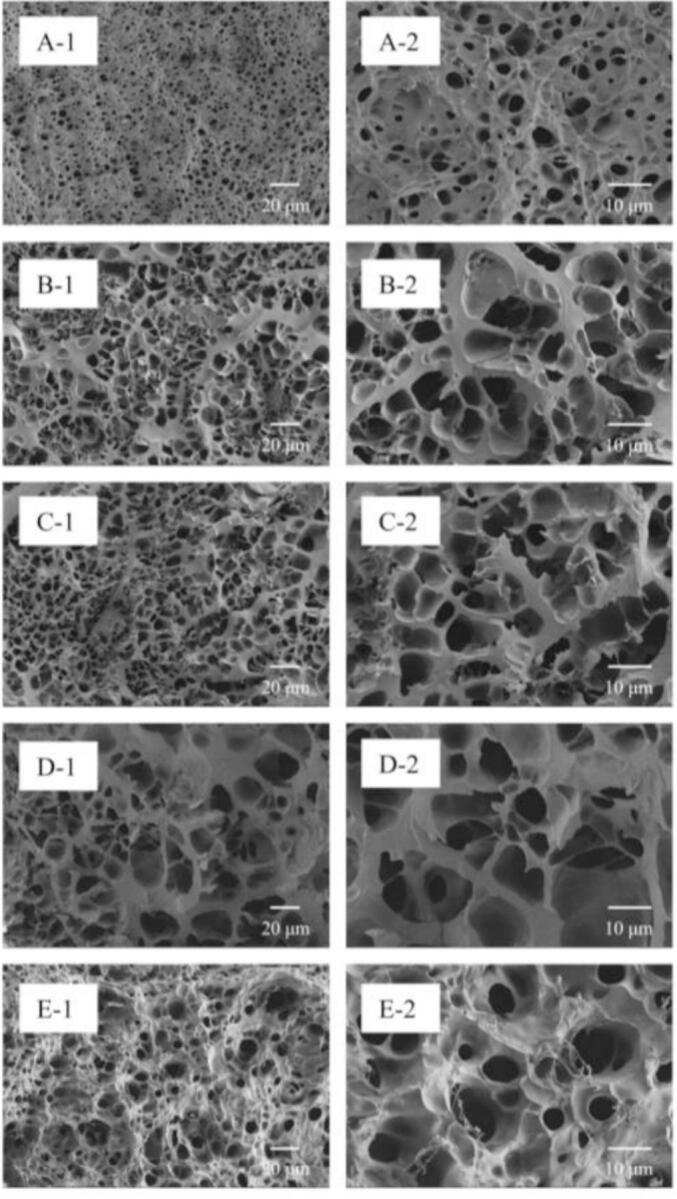
Scanning electron micrographs of rice balls with the addition of different spirulina powder concentrations (500× and 1500×). A: rice balls without spirulina powder addition; B: rice balls with 1% spirulina powder addition; C: rice balls with 3% spirulina powder addition; D: rice balls with 5% spirulina powder addition; E: rice balls with 7% spirulina powder addition.

The SEM micrographs illustrating the microstructural changes in rice balls formulated with different concentrations of spirulina powder are depicted in Fig. 5. After steaming and thermal treatment, the cross-sections of rice balls exhibited a porous structure due to moisture diffusion and starch gelatinization. With increasing spirulina powder concentrations, the pores on the grain surface and in the cross-sections became progressively larger and more numerous, indicating that spirulina incorporation disrupted the originally continuous microstructure of the rice matrix; a similar phenomenon was also reported by Batista et al. (2019). This microstructural change was consistent with the LF-NMR results, particularly the increase in T_22_ and the rebound of A_23_ at 7% spirulina addition, suggesting that the enlarged pore structure provided more pathways for water migration and reduced the local immobilization of water. Niu et al. (2017) similarly reported that additives can alter the pore structure of starch gels, thereby affecting water binding and moisture migration. This finding may be attributed to the retrogradation process, during which spirulina powder interferes with the rearrangement and reassociation of starch molecular chains, weakens the interactions between water and starch chains, and thereby promotes water migration and syneresis (Zhang et al., 2019). During steaming and thermal treatment, the spirulina cell wall was disrupted, releasing hydrophilic constituents such as proteins, polysaccharides, and dietary fiber. These components adsorbed part of the free water, thereby contributing to the enlargement of pores in the rice ball matrix (Bürck et al., 2024). Moreover, adding excessive spirulina concentrations may increase the structural disorder of the gel network (Wang et al., 2023). When the spirulina powder concentration reached 7%, distinct filamentous structures were detected on the pore–wall surface (Fig. 5E). This finding may be related to the enrichment of soluble proteins and polysaccharides released from spirulina during steaming and thermal treatment at the pore edges, which subsequently formed a viscoelastic continuous phase.

### 3.8 Analysis of volatile flavor compounds

**Fig. 6:**
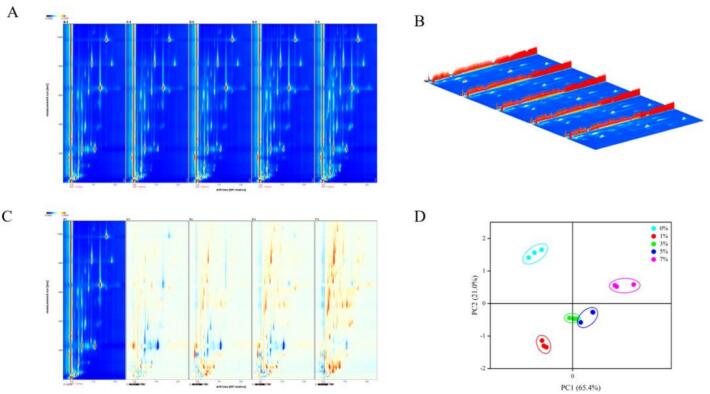
GC-IMS two-dimensional top-view plot (A), three-dimensional spectrum (B), pseudocolor difference plot (C), and PCA plot (D) of rice balls with the addition of different concentrations of spirulina powder.

GC-IMS was used to analyze the volatile organic compounds (VOCs) in the rice balls containing five different concentrations of spirulina powder. In the two-dimensional spectra (Fig. 6A), the overall peak positions were largely consistent among groups, suggesting a similar primary spectral framework of volatile constituents. Nevertheless, differences in the signal intensities of multiple characteristic peaks and in the local band density were evident, indicating that spirulina incorporation can markedly alter the relative abundance distribution of volatile flavor compounds. Fig. 6B depicts the three-dimensional spectra generated by GC-IMS, where variations in peak intensities indicate differences in VOC profiles among the samples. Multiple volatile signal peaks were detected at identical retention time and drift time coordinates across samples containing different concentrations of spirulina powder (Fig. 6B), indicating a relatively rich repertoire of volatile components in the rice ball matrix. Furthermore, the volatile chemical profiles exhibited visible variations among samples with changes in the spirulina powder concentrations. The difference map (Fig. 6C) further demonstrated that, with increasing spirulina concentrations, the signals of multiple characteristic peaks increased (red), indicating elevated concentrations of certain VOCs. In the PCA plot (Fig. 6D), PC1 (65.4%) and PC2 (21.0%) jointly explain 86.4% of the total variance. Samples containing different spirulina concentrations formed distinct clusters with good within-group aggregation, indicating that spirulina incorporation induces significant and reproducible overall differences in the volatile flavor composition of the rice balls. These results indicate that spirulina addition mainly changed the relative abundance of characteristic VOCs within a generally similar volatile profile, and this effect became more pronounced with increasing addition levels.

**Fig. 7:**
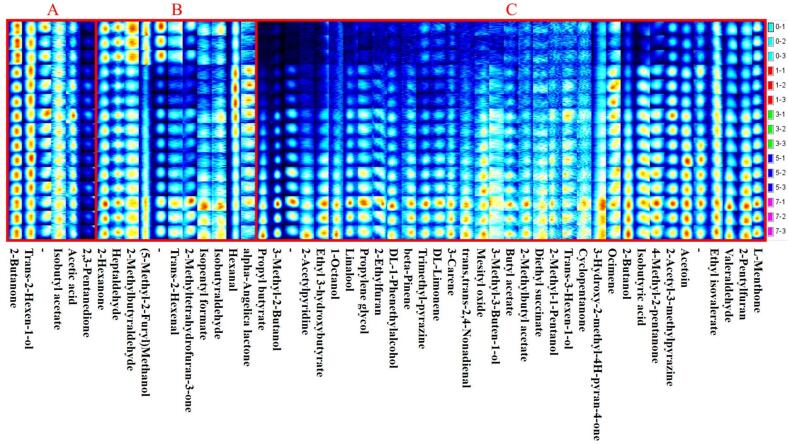
GC-IMS fingerprint of rice balls containing different concentrations of spirulina powder.

A total of 52 volatile compounds were identified in the spirulina-enriched rice balls, including 12 alcohols, 9 esters, 7 aldehydes, 9 ketones, 4 alkenes, 2 furans, 2 pyrazines, 1 pyridine, and 2 acids, along with 4 unidentified compounds (Fig. 7 and Table 7). The major aroma-active compounds in cooked rice are primarily aldehydes, alcohols, ketones, and heterocyclic compounds (Ye et al., 2024). Spirulina inherently contains diverse classes of VOCs, commonly including aldehydes, ketones, alcohols, esters, terpenes/hydrocarbons, furans, and pyrazines (Paraskevopoulou et al., 2024). Fig. 7 also shows that, with increasing spirulina concentrations, the concentrations of certain volatile flavor compounds increase (Region C). Furthermore, spirulina contains a certain amount of lipids and unsaturated fatty acids, which are susceptible to lipid oxidation under heating and oxygen exposure, thereby generating or increasing aldehydes and other oxidation-derived volatiles. Among these, hexanal is widely considered an important marker of secondary lipid oxidation (Grebenteuch et al., 2021); Region B is also presented in this format. Therefore, the increase in aldehyde-related signals may contribute to the stronger green, grassy, fatty, or algal-like notes of spirulina-enriched rice balls. In addition to aldehydes, the detection of 2-acetyl-3-methylpyrazine, trimethyl-pyrazine, and 2-acetylpyridine suggests that nitrogen-containing thermal reaction products also contributed to the volatile profile. Pyrazines are typical Maillard reaction-derived compounds and generally contribute roasted, nutty, toasted, or popcorn-like aroma characteristics (Yu et al., 2021). Therefore, the increased protein content caused by spirulina addition may provide more amino-containing precursors for Maillard-type reactions during steaming, thereby contributing to the formation of heterocyclic aroma compounds.

**Table 7:** Qualitative analysis of volatile compounds in rice balls.

Category	NO.	Volatile compounds	CAS	Formula	MW	RI	Rt[s]	Dt[ms]	Flavor description
Alcohols	1	Trans-2-hexen-1-ol	C2305217	C_6_H_12_O	100.2	867.5	201.905	1.1848	fruity green leafy
	2	Propylene glycol	C57556	C_3_H_8_O_2_	76.1	737	82.921	1.2623	odorless very slight alcoholic
	3	3-Methyl-3-buten-1-ol	C763326	C_5_H_10_O	86.1	738.4	83.815	1.2778	sweet fruity
	4	3-Hydroxy-2-methyl-4H-pyran-4-one	C118718	C_6_H_6_O_3_	126.1	1087	605.265	1.1538	sweet caramel jam fruity baked bread
	5	2-Butanol	C78922	C_4_H_10_O	74.1	1052.2	516.166	1.1488	sweet apricot
	6	Linalool	C78706	C_10_H_18_O	154.3	1110.8	674.755	1.2425	citrus floral sweet bois
	7	DL-1-phenethylalcohol	C98851	C_8_H_10_O	122.2	1057.6	529.194	1.5659	fresh sweet gardenia hyacinth
	8	Trans-3-hexen-1-ol	C928972	C_6_H_12_O	100.2	844.5	173.569	1.5386	leafy petal oily earthy
	9	3-Methyl-2-butanol	C598754	C_5_H_12_O	88.1	680.1	56.008	1.2397	fruity
	10	(5-Methyl-2-furyl)methanol	C3857258	C_6_H_8_O_2_	112.1	951.1	319.065	1.2604	sweet caramellic
	11	1-Octanol	C111875	C_8_H_18_O	130.2	1052.4	516.648	1.471	green orange aldehydic rose mushroom
	12	2-Methyl-1-pentanol	C105306	C_6_H_14_O	102.2	843.2	172.018	1.5863	–
Esters	13	Isopentyl formate	C110452	C_6_H_12_O_2_	116.2	790.5	121.583	1.2675	plum vinous fruit green notes
	14	Ethyl isovalerate	C108645	C_7_H_14_O_2_	130.2	1082	591.441	1.2506	sweet, fruity
	15	Propyl butyrate	C105668	C_7_H_14_O_2_	130.2	1156.3	830.561	1.268	apricot, pineapple
	16	Alpha-angelica lactone	C591128	C_5_H_6_O_2_	98.1	861.5	194.12	1.1217	sweet, creamy, coconut
	17	Isobutyl acetate	C110190	C_6_H_12_O_2_	116.2	984	376.598	1.2383	sweet fruity ethereal banana tropical
	18	Butyl acetate	C123864	C_6_H_12_O_2_	116.2	789.6	120.826	1.2315	ethereal solvent fruity banana
	19	Ethyl 3-hydroxybutyrate	C5405414	C_6_H_12_O_3_	132.2	935.7	295.314	1.1727	green, fruity and winey
	20	2-Methylbutyl acetate	C624419	C_7_H_14_O_2_	130.2	1124.5	718.217	1.2948	sweet, banana, fruity, estry
	21	Diethyl succinate	C123251	C_8_H_14_O_4_	174.2	1194.8	990.142	1.2966	mild fruity cooked apple ylang
Aldehydes	22	2-Methylbutyraldehyde	C96173	C_5_H_10_O	86.1	887	229.66	1.3955	musty fusel rummy nutty
	23	Valeraldehyde	C110623	C_5_H_10_O	86.1	667.6	52.114	1.1827	winey, fermented, bready
	24	Isobutyraldehyde	C78842	C_4_H_8_O	72.1	790.5	121.578	1.2889	fresh aldehydic floral green
	25	Hexanal	C66251	C_6_H_12_O	100.2	1060	535.082	1.2537	green, woody, vegetative, apple
	26	Heptaldehyde	C111717	C_7_H_14_O	114.2	885.8	227.839	1.3431	green aldehydic oily cortex grassy
	27	Trans-2-hexenal	C6728263	C_6_H_10_O	98.1	884.7	226.071	1.5288	green banana aldehydic fatty cheesy
	28	trans,trans-2,4-Nonadienal	C5910872	C_9_H_14_O	138.2	1204.9	1036.947	1.3538	fatty waxy chicken fat citrus rind
Ketones	29	2-Butanone	C78933	C_4_H_8_O	72.1	892.7	237.76	1.2575	acetone-like ethereal fruity camphor
	30	2-Methyltetrahydrofuran-3-one	C3188009	C_5_H_8_O_2_	100.1	784.4	116.453	1.4165	sweet solvent bread buttery nutty
	31	2-Hexanone	C591786	C_6_H_12_O	100.2	789.9	121.124	1.1829	fruity fungal meaty buttery
	32	4-Methyl-2-pentanone	C108101	C_6_H_12_O	100.2	737.9	83.499	1.1736	green, vegetative, herbal, fruity
	33	2,3-Pentanedione	C600146	C_5_H_8_O_2_	100.1	610.5	37.452	1.228	creamy caramel nutty cheese
	34	Acetoin	C513860	C_4_H_8_O_2_	88.1	690.8	59.61	1.058	sweet buttery creamy dairy milky fatty
	35	l-menthone	C14073973	C_10_H_18_O	154.3	1133.3	747.8	1.336	cooling, peppermint, fresh green
	36	Cyclopentanone	C120923	C_5_H_8_O	84.1	781.5	113.986	1.3314	minty
	37	Mesityl oxide	C141797	C_6_H_10_O	98.1	1112.6	680.359	1.1279	pungent earthy vegetable acrylic
Alkenes	38	3-Carene	C13466789	C_10_H_16_	136.2	1144.1	785.647	1.2199	citrus pine terpenic herbal
	39	Ocimene	C13877913	C_10_H_16_	136.2	1059.6	533.998	1.6748	green, tropical, woody with floral
	40	DL-limonene	C138863	C_10_H_16_	136.2	1194.8	990.142	1.2966	citrus herbal terpene camphor
	41	Beta-pinene	C127913	C_10_H_16_	136.2	1124.5	718.408	1.2178	dry woody resinous pine hay green
Furans	42	2-Pentylfuran	C3777693	C_9_H_14_O	138.2	986.9	382.224	1.2589	fruity green earthy beany vegetable
	43	2-Ethylfuran	C3208160	C_6_H_8_O	96.1	736.4	82.587	1.3114	cocoa bready malty coffee
Pyrazines	44	2-Acetyl-3-methylpyrazine	C23787806	C_7_H_8_N_2_O	136.2	1086.4	603.6	1.1731	nutty nut hazelnut toasted grain
	45	Trimethyl-pyrazine	C14667551	C_7_H_10_N_2_	122.2	984.9	378.302	1.6244	raw nut skin vegetable cocoa
Pyridines	46	2-Acetylpyridine	C1122629	C_7_H_7_NO	121.1	1038.5	484.91	1.1191	popcorn heavy corn chip fatty tobacco
Acids	47	Isobutyric acid	C79312	C_4_H_8_O_2_	88.1	748.3	89.927	1.1602	acidic sour cheese dairy buttery rancid
	48	Acetic acid	C64197	C_2_H_4_O_2_	60.1	665.8	51.58	1.3344	sharp pungent sour vinegar

Note: “-” implies not detected.

## 4 Conclusion

Spirulina powder was incorporated into rice and processed by cooking to produce spirulina-enriched rice balls, which exerted significant impacts on the nutritional composition, color attributes, physicochemical properties, structural characteristics, and volatile flavor compounds of the rice balls. Compared with that of control rice balls, the incorporation of spirulina improved the nutritional value of the product, as evidenced by increased contents of protein and fat and a concomitant reduction in carbohydrate content, indicating a certain nutrient-fortification effect and practical application value in the development of nutrient-enriched rice-based products. At low spirulina concentrations, the water distribution shifted toward a higher proportion of bound water and a lower proportion of free water, suggesting that the hydrophilic constituents in spirulina contributed to water immobilization; moreover, the short- and long-range order of starch improved. Considering the concentration-dependent effects, 3%–5% spirulina addition may be considered a preliminary suitable range for rice ball production, as this range could improve nutritional value while maintaining relatively favorable structural properties. Nevertheless, spirulina incorporation caused an overall green coloration that intensified with increasing addition levels, and the hardness of the rice balls also increased significantly, resulting in a generally more compact texture. At a high concentration (7%), the rice ball matrix tended to become looser with a reduced water-holding capacity; the SEM observations revealed enlarged and more numerous pores, indicating a remarkable alteration in the original microstructural network. Spirulina incorporation also reshaped the profile of volatile flavor compounds in the rice balls, with significant differences found across addition concentrations. Although this may increase flavor diversity, it could also introduce odor attributes that are less acceptable to certain consumers. Overall, spirulina-enriched rice balls show advantages in nutritional enhancement, product diversification, and value-added utilization of rice-based foods, while excessive spirulina addition may adversely affect texture, water retention, microstructure, and aroma acceptability. The present study mainly focused on the physicochemical, structural, and volatile properties of spirulina-enriched rice balls, while sensory evaluation, consumer acceptability, storage stability, and flavor optimization were not fully investigated. Future studies should comprehensively consider sensory evaluation, consumer acceptability, storage-stability assessment, and flavor optimization to further screen and determine a more appropriate addition range for practical application and product development.

## CRediT authorship contribution statement

**Xiwu Jia:** Writing – review & editing, Funding acquisition, Conceptualization. **Yujun Shi:** Writing – original draft, Visualization, Investigation. **Zhili Ji:** Visualization, Supervision. **Wangyang Shen:** Supervision, Resources. **Xin Liu:** Writing – review & editing, Supervision. **Bo Yu:** Methodology, Conceptualization. **Yolani Syaputri:** Writing – review & editing. **Hongjian Zhang:** Methodology, Data curation.

## Funding sources

Hubei Provincial Science and Technology Department (2025BBB062), Hainan Provincial Science and Technology Special Envoys Programme (KJTP202553 and KJTP202552), Science and Technology Talent Service Enterprise Project of Hubei Province (2024DJC056), and 10.13039/501100008960Wuhan Polytechnic University (2025Y40) for financial support.

## Declaration of competing interest

The authors declare that they have no known competing financial interests or personal relationships that could have appeared to influence the work reported in this paper.

## Data Availability

Data will be made available on request.
